# When touch is stressful: acute endocrine and behavioral responses of domestic rabbits to unfamiliar human handling

**DOI:** 10.3389/fvets.2026.1793812

**Published:** 2026-03-06

**Authors:** Michaela Součková, Martina Frühauf Kolářová, Lucie Přibylová, Katarína Kováčová, Michal Zeman

**Affiliations:** 1 Department of Ethology and Companion Animal Science, Faculty of Agrobiology, Food and Natural Resources, Czech University of Life Sciences Prague, Prague, Czechia; 2 Department of Veterinary Sciences, Faculty of Agrobiology, Food and Natural Resources, Czech University of Life Sciences Prague, Prague, Czechia; 3 Department of Animal Physiology and Ethology, Faculty of Natural Sciences, Comenius University Bratislava, Bratislava, Slovakia

**Keywords:** animal-human interaction, behavioral observation, corticosterone, rabbit, stress

## Abstract

**Background:**

Rabbits are increasingly kept as companion animals, yet little is known about their stress responses during interactions with unfamiliar humans—situations commonly encountered during household visits or animal-assisted interventions. This study evaluated whether tactile interaction with an unfamiliar person induced acute stress in domestic rabbits using physiological (salivary corticosterone) and behavioral indicators (ear position, eye openness, and body posture).

**Methods:**

Seven adult, intact female dwarf rabbits were each exposed five times to a 10-min stroking session while sitting on an unfamiliar person’s lap, simulating a typical human– rabbit interaction. Salivary corticosterone was measured under control conditions (no stroking) and experimental conditions (20 min post-interaction), while behavior was recorded during the stroking period.

**Results:**

Tactile interaction with an unfamiliar person resulted in a significant increase in corticosterone concentrations (mean +214.4 ± 74.1%, *p* = 0.031). Behaviorally, rabbits spent an average of 8.4 min in a tense posture, held their ears pressed back for 4.2 min, and kept their eyes partially or fully closed for 0.7 min. Tense posture in rabbits significantly correlated (*r* = 0.82; *p* = 0.03) with increased corticosterone levels; moreover, a tendency toward a correlation (*p* = 0.088) between ears pressed back and increased corticosterone levels was observed.

**Conclusion:**

These results indicate that handling by an unfamiliar person elicits acute stress responses in rabbits and should be considered when interacting with rabbits.

## 1 Introduction

The popularity of rabbits as companion animals has increased recently (1–3). Moreover, rabbits are being increasingly used in animal-assisted interventions (AAI) and animal-assisted services (AAS) (4). However, the welfare of rabbits and possible stress caused by interactions with unfamiliar humans in the context of animal-assisted interventions has received only limited research attention (5–7), with one questionnaire study conducted in domestic environments (8). Moreover, in this context, previous research focused only on behavioral indicators (5–7).

To evaluate the welfare-related effects, it is essential to identify reliable indicators of stress. In rabbits, behavioral manifestations are among the most accessible and informative measures, as they reflect evolutionarily conserved defense strategies and provide valuable insights into the level of physiological load (9). In rabbits, behavioral indicators of stress include alterations in ear position (10–12) and facial expressions, such as eye closure or partial narrowing of the eyes (5, 13). Changes in grooming behavior (10, 14) are dependent on the situation. Among defensive strategies as flight, fight, or freeze response (15–17), rabbits typically rely on flight as the primary defense; however, when escape is impossible, they usually adopt immobility (5) rather than aggression. This immobility or freezing response may easily be misinterpreted as an absence of stress (18, 19). These behaviors represent natural reactions to aversive stimuli and, although their interpretation may involve a degree of subjectivity, they provide valuable insights into the rabbit’s cognitive and emotional state (5). In addition to facial and activity-related changes, postural displays are also key indicators of emotional responses.

On the other hand, physiological indicators represent a central and inseparable component in the studies of stress in animals (20, 21). A primary physiological marker of the stress response in rabbits is corticosterone (22), the principal glucocorticoid in many other animal taxa, including birds (23) and rodents (24). Corticosterone levels reflect activation of the hypothalamic-pituitary-adrenal (HPA) axis and provide a reliable indicator of both acute and chronic stress (25). Quantifying this hormone yields objective, quantitative information about an organism’s physiological response to stressors (26), which is essential for evaluating stress-related body reactions. Physiological sampling methods vary in their degree of invasiveness. Blood sampling for corticosterone analysis represents an invasive procedure, and the handling involved can itself elicit stress, thereby influencing hormone levels within minutes (27–29). Consequently, increasing attention has been directed toward non-invasive techniques that minimize such effects and are now widely applied (30). Among the most commonly used non-invasive methods for assessing stress are analyses of corticosterone concentrations in feces, saliva, and hair samples (31–33). While blood and saliva samples are typically used to evaluate acute stress (34), feces (35) and hair (36, 37) are primarily employed to assess chronic stress.

To date, studies combining behavioral and physiological parameters during human–animal interaction have only been systematically documented in dogs (38) and horses (39). These studies demonstrate that reliable assessment of stress responses requires integration of both behavioral and physiological measures. Behavioral observations alone may be prone to subjectivity and misinterpretation (40, 41), while physiological indicators can be influenced by numerous internal and external factors (42) such as emotional arousal, physical activity (43), or inappropriate sampling procedures (44).

This multi-level approach is essential for understanding stress responses in domestic rabbits; however, it has not yet been systematically investigated. This gap considerably limits the ability to objectively assess rabbit welfare. Therefore, the present study aimed to experimentally examine whether there are any signs of acute stress (increased corticosterone level and stress-related behavior) induced by commonly used tactile interaction with an unfamiliar human—specifically, when a rabbit is held on a person’s lap. Moreover, we aimed to investigate whether corticosterone level correlates with behavioral indicators of the stress response.

## 2 Materials and methods

### 2.1 Study design

**Figure 1:**
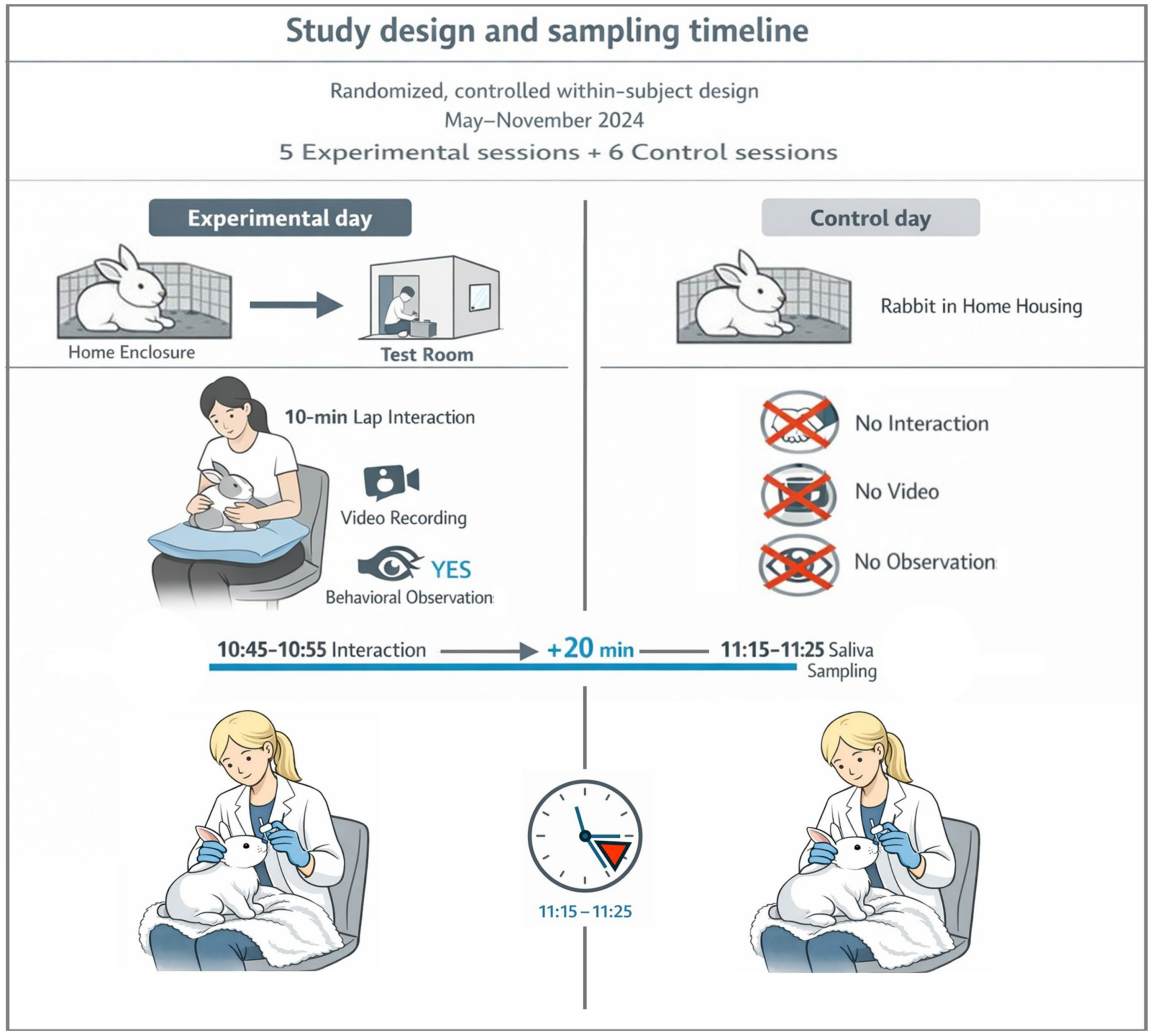
Visual methodology.

The experiment was conducted from May to November 2024 under consistent conditions regarding room arrangement, ambient temperature (20 °C), and time of day. Experimental recordings were always conducted between 10:45 and 10:55 a.m. CET, corresponding to the circadian rhythm phase of rabbits characterized by stable activity levels (i.e., neither at peak nor minimum activity) (45–47). The methodology combined measurements of salivary corticosterone with behavioral assessment following exposure to stroking by an unfamiliar person. On experimental days, behavioral observation was performed first and corticosterone sampling afterward, whereas on control days only corticosterone samples were collected to establish baseline corticosterone levels of the rabbits. For visual methodology see Figure 1.

Each rabbit experienced the same experimental situation five times, with saliva collected after each session, and control saliva samples were collected six times on separate days.

**Figure 2:**
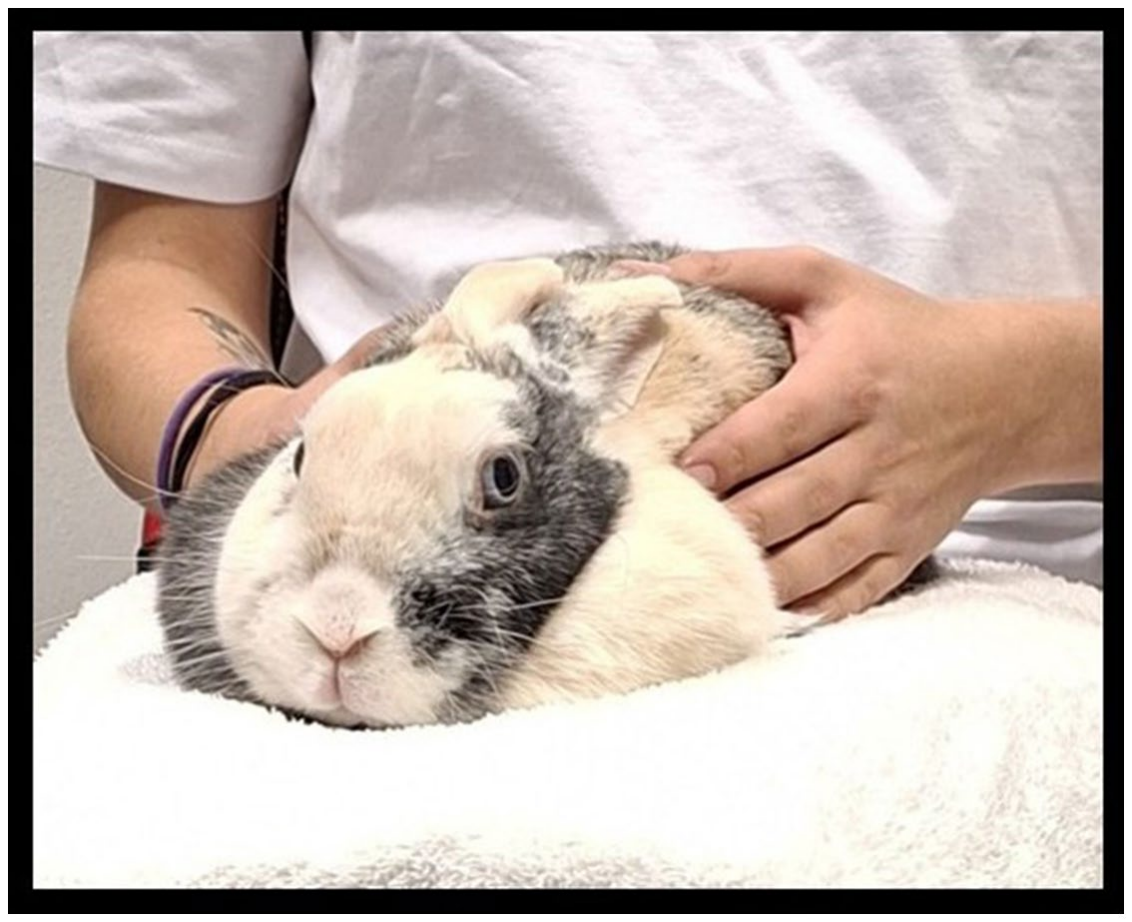
Interaction with a rabbit on the lap of an unfamiliar person.

The experimental observation consisted of a 10-min tactile interaction on the lap of an unfamiliar person (see Figure 2). To minimize transport-related stress, the procedure took place in a room adjacent to the housing area. A familiar person (2 caregivers) carried the rabbit to the test room, placed it on a cushion on the participant’s lap for 10 min, and then returned it to the home enclosure. Participants were instructed to remain calm, avoid sudden sounds or movements, and gently stroke the rabbit’s body while avoiding the head. Before each session, participants changed into standardized clothing (white T-shirt, black trousers). All textile materials (participants’ clothes and cushion covers) were washed at high temperature after each use with *Desilinie DC* detergent (1 L), which has bactericidal, fungicidal, and virucidal properties and is odor-free. The washed textiles were subsequently stored away from the rabbits.

A saliva sample was collected 20 min after the tactile interaction with an unfamiliar person by one of the familiar caregivers (who also served as an experimenter) for corticosterone quantification. This post-interaction interval was selected for the present pilot study by analogy with canine research, where salivary cortisol is commonly sampled following standardized human handling/interaction paradigms to capture the delayed endocrine response in saliva (48, 49). In rabbits, however, no published evidence was found empirically validating a comparable post-interaction sampling window for salivary corticosterone (or the corresponding primary glucocorticoid). To allow direct comparison with control days, both experimental and control corticosterone sampling were always collected between 11:15 and 11:25 a.m. The control samples were collected on days when rabbits were not handled. They were taken from their home enclosure by a familiar caretaker and placed on the caretaker’s lap within the same room.

### 2.2 Participants of the study

#### 2.2.1 Rabbits

A total of seven intact female dwarf domestic rabbits (*Oryctolagus cuniculus f. domesticus*) were included in the study. All rabbits were fully socialized and accustomed to regular human contact with familiar and unfamiliar people (both children and adults). Initial socialization occurred with their respective breeders until eight weeks of age; from that age onwards, all individuals were housed together under identical conditions resembling a standard home environment. They were in daily contact with both adults and children and were routinely exposed to common household stimuli. At the time of inclusion in the experiment, rabbits were 6.0–6.1 years old (mean age: 6.05 years). All animals were clinically healthy, in good body condition, and underwent regular veterinary examinations.

The rabbits were housed in three sibling groups (2 + 2 + 3 individuals). Each group was kept separately in enclosures measuring 215/180 × 115 × 90 cm, equipped with ample environmental enrichment (hiding places, toys, tunnels, and feeding enrichment such as branches, twigs, and hay-based items). Each group also had access to a secured outdoor run with an area of 7 m^2^, which they used for at least 8 h per day outside the enclosure.

Within their housing units, rabbits had ad libitum access to hay and water. Their diet was supplemented regularly with pelleted complete feed, fresh fruits, vegetables, and herbs to enrich their nutritional intake.

The use of animals in this study was approved by the Ethics Committee for the Welfare of Experimental Animals of the Czech University of Life Sciences Prague (approval number PP0220) as part of a broader research project.

#### 2.2.2 Human participants

Female participants aged 18–35 years (mean age: 24.9 years; hereafter referred to as “participants”) took part in the study. Participants were students and employees of the Czech University of Life Sciences Prague, recruited in person by the experimenters. Each participant was invited to attend the experimental recording at a scheduled time in the room where the rabbits were housed and where the experiment took place. Participation in the study was voluntary, and no financial or material compensation was provided.

All participants were informed in advance about the aims and conditions of the project and signed a written informed consent form. The consent also included agreement to the 10-min video recording of the rabbit–human interaction. Participants were instructed on occupational safety procedures and informed about possible adverse reactions of animals, such as scratching or biting. Each participant had the right to withdraw from the study at any stage without providing a reason.

Personal data and recordings of the participants were accessible only to the researchers conducting the experiment. All collected data were processed and analyzed anonymously.

All procedures involving human participants were carried out in accordance with the Declaration of Helsinki and were approved by the Ethics Committee of the Czech University of Life Sciences Prague (license number 052022/25).

### 2.3 Determination of salivary corticosterone concentration

#### 2.3.1 Saliva sampling

Saliva samples were collected on both the control and experimental days under identical conditions. Sampling was carried out in the room where the rabbits were permanently housed. To minimize stress, samples were collected by the rabbits’ caregivers while the rabbits were sitting on their laps. No food stimulation was provided before or during sampling.

Immediately after collection, samples were centrifuged for 20 min at 5,000 rpm (Hettich Zentrifuge Universal 32R, Andreas Hettich GmbH, Tuttlingen, Germany). The supernatant was separated and stored at −80 °C until analysis.

Saliva was collected using Salivette^®^ swabs (Sarstedt), with each swab trimmed to match the oral cavity size of a dwarf rabbit. Each swab was attached to a thin metal wire to secure it during sampling and prevent swallowing or mechanical damage. The swab was placed in the oral cavity for 3–4 min as recommended by Kobelt et al. (50) and gently moved to stimulate salivary glands and maximize saliva production. Control sampling was conducted over one month, this approach allowed hormonal changes to be evaluated relative to each individual’s own physiological range rather than relying solely on absolute values, due to substantial individual differences in glucocorticoid stress responses, which are influenced by genetic factors, life experiences, and stable behavioral styles (51, 52). The total number of control samples was 42 (6 per rabbit), and a total of 35 experimental samples were obtained (5 per rabbit) (see Supplementary Table S1).

#### 2.3.2 Analytical determination of corticosterone concentration

Corticosterone concentrations in rabbit saliva were determined using a commercial enzyme immunoassay kit (Corticosterone ELISA Kit, Cayman Chemical, Ann Arbor, MI, United States) following the manufacturer’s protocol. This competitive immunoassay is designed for the quantification of corticosterone in serum, feces, and other biological matrices. The assay has a working range of 8.2–5,000 pg/mL and a sensitivity of approximately 30 pg/mL at 80% B/B₀.

Absorbance was measured after 90–120 min of incubation at a wavelength of 412 nm using an Epoch 2 Microplate Spectrophotometer (Agilent, BioTek Instruments, Winooski, VT, United States). All samples were analyzed in one assay at a 1:100 dilution, in duplicates, following the standardized procedure recommended by the manufacturer (Cayman Chemical).

### 2.4 Behavioral observation

Behavioral observations were conducted on experimental days during the tactile interaction with an unfamiliar person. After the unfamiliar person was seated and the rabbit was placed on a cushion on their lap, a 10-min video recording was started.

Rabbit behavior was recorded using the rear camera of a Samsung Galaxy A54 5G smartphone (50 MP Quad Bayer sensor) positioned frontally to capture the behavior of the rabbit. A total of 35 video recordings were obtained. Continuous recording and simultaneous analysis by two trained observers were performed using The Observer XT software (Noldus). This approach minimized potential errors in behavioral coding. All recordings were coded concurrently by both observers, and inter-observer agreement was required to be 100%. If any disagreement between the observers occurred, the rabbits’ behavior was carefully discussed until consensus was reached.

The duration of the following behavioral indicators was selected based on a literature review using databases Web of Science, Google Scholar, PubMed, and ScienceDirect with the following keywords: *rabbit, stress, Oryctolagus cuniculus f. domesticus, behavior, welfare, grimace, body posture, tense, freezing* (5, 10, 53–59) to reflect the rabbits’ affective states: ear position, eye openness, and body configuration.

**Figure 3:**
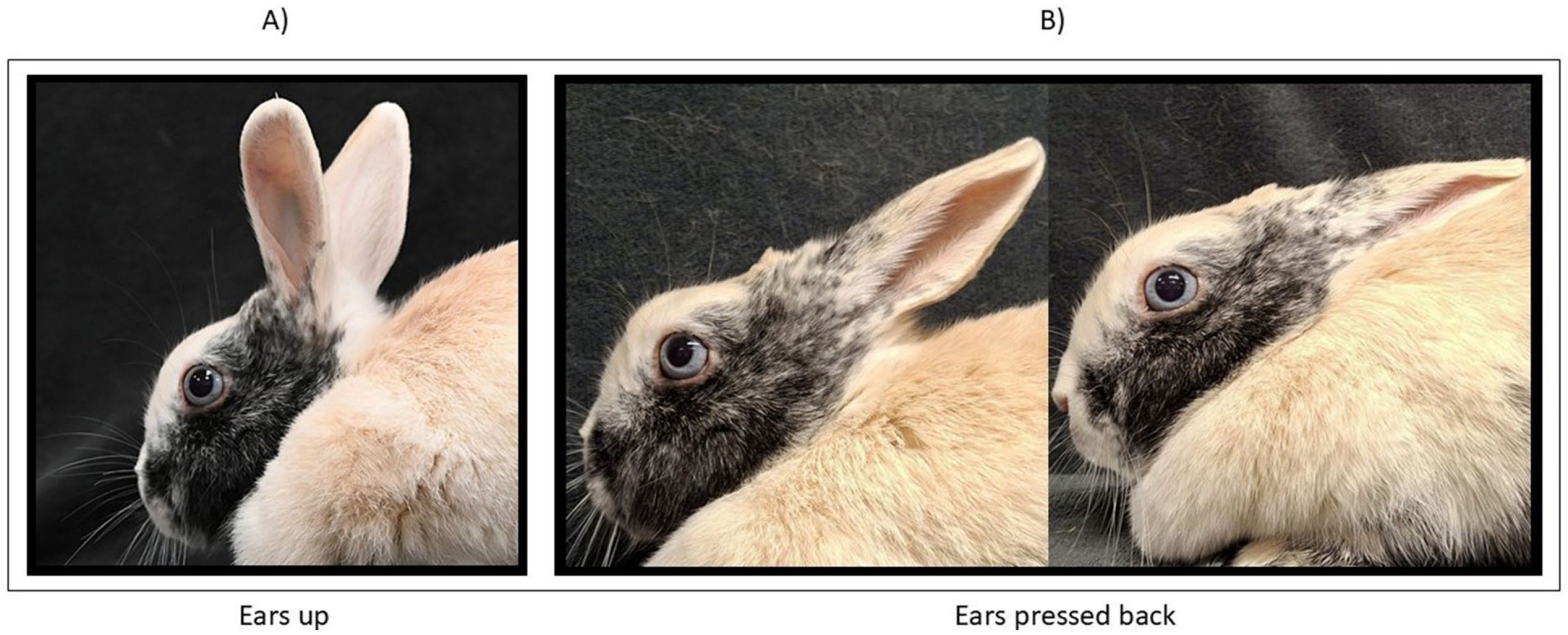
Ears positions. **(A)** upright, indicationg no threatening; **(B)** flattened or laid back, indicating discomfort.

Ears were coded in two positions: (1) upright, indicating no threatening (see Figure 3A); (2) flattened or laid back, indicating discomfort (see Figure 3B).

**Figure 4:**
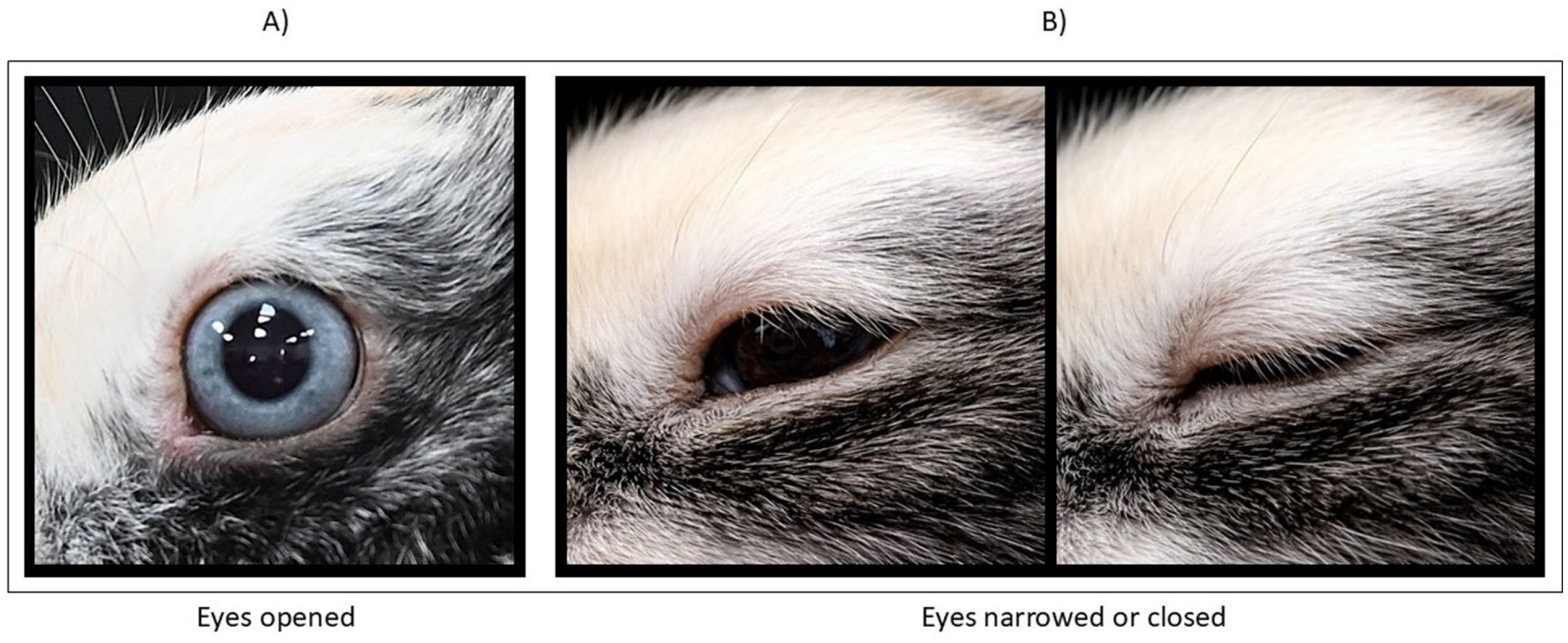
Eyes positions. **(A)** open, indicating relaxation; **(B)** narrowed or closed, indicating discomfort.

Eyes were coded in two positions: (1) open, indicating relaxation (see Figure 4A); (2) narrowed or closed, indicating discomfort (see Figure 4B).

**Figure 5:**
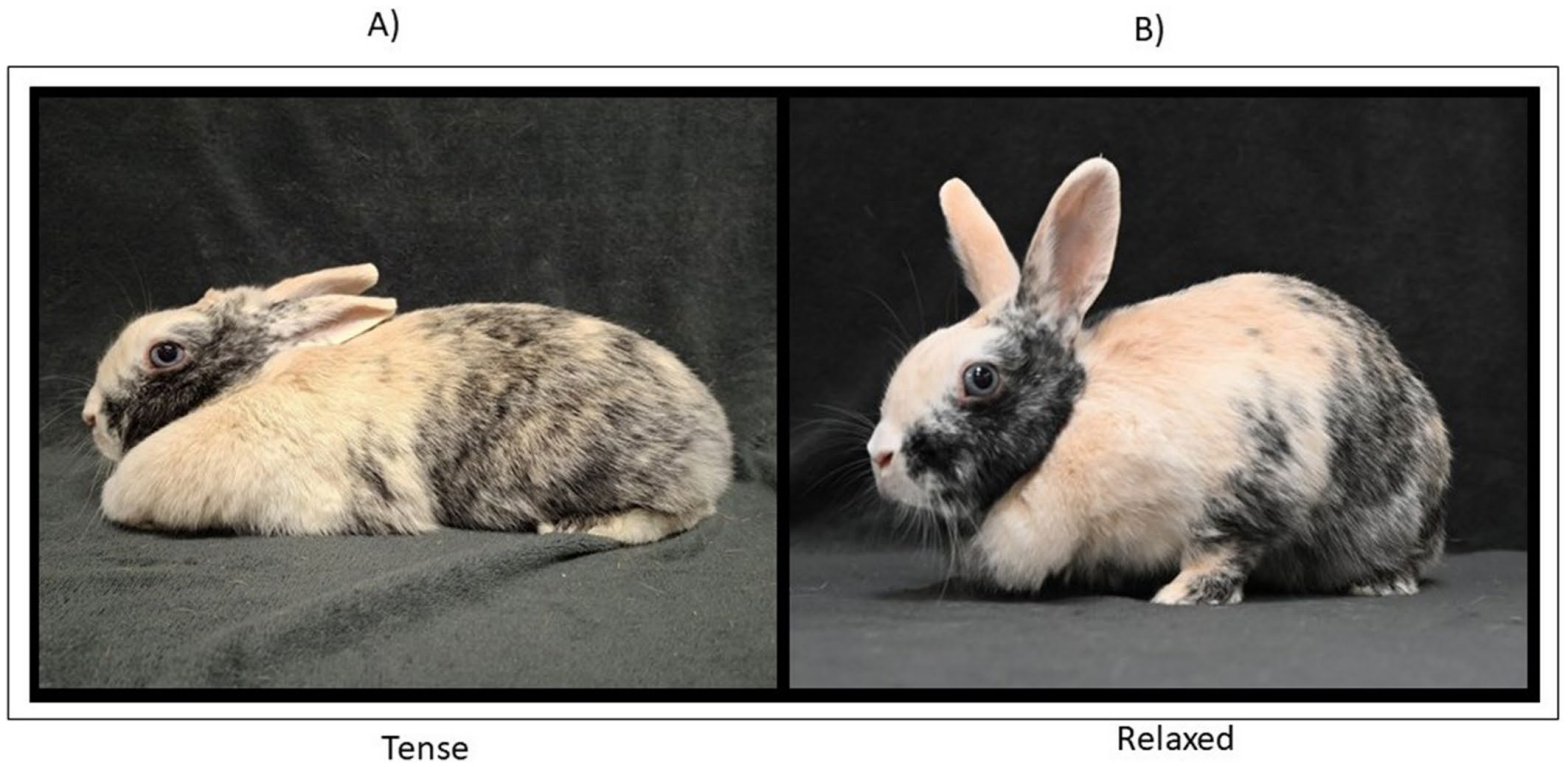
Body configuration. **(A)** tense, characterized by rigidity and lack of visible relaxation (labeled “Tense”, **B**) relaxed, characterized by absence of vigilance.

Body configuration was coded as: (1) tense, characterized by rigid posture and lack of visible relaxation (see Figure 5A); (2) relaxed with lack of vigilance (see Figure 5B).

All behaviors were coded as duration variables.

### 2.5 Statistical analysis

Statistical analyses and graphs were conducted using GraphPad Prism version 9.5.1 (San Diego, CA, United States). Data normality was assessed using the Shapiro–Wilk test. Statistical analyses combined both descriptive and inferential approaches to account for both individual variability and group-level effects.

Differences in corticosterone concentration between control and experimental conditions were evaluated using the Wilcoxon signed-rank test. To quantify changes in corticosterone under experimental conditions relative to control, the mean corticosterone concentration during the control condition was normalized to 100%, and experimental values were expressed as percentage change relative to control. Results are presented as mean ± SEM.

Behavioral data consisted of five 10-min observation sessions per rabbit. Differences across repeated observation sessions were therefore assessed using the Friedman test for repeated measures. In cases with missing data, a mixed effect model was applied. Individual differences between animals were described descriptively to illustrate inter-individual variability.

Correlation between percentage changes in corticosterone levels and behavioral parameters were assessed using Spearman’s rank correlation.

## 3 Results

### 3.1 Corticosterone response of the rabbits to tactile contact with an unfamiliar person

**Figure 6:**
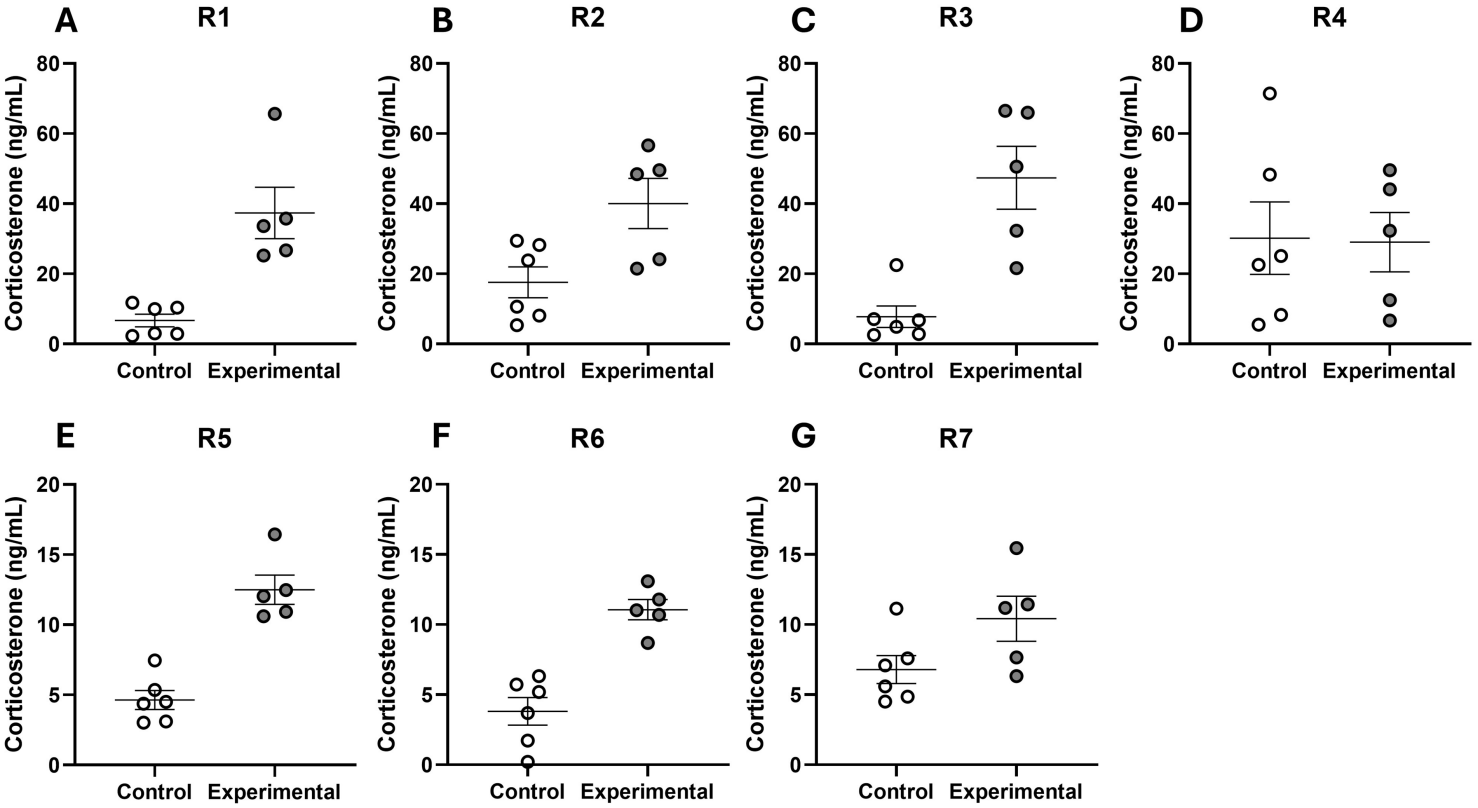
Individual corticosterone concentrations. Panels **A–G** correspond to individual rabbits (*n* = 7; R1–R7). Values are presented as absolute corticosterone levels (ng/mL) for control (white dots; *n* = 6) and experimental conditions (grey dots; *n* = 5). Note that the Y-axes are scaled differently: the upper panels range up to 80 ng/mL, while the lower panels range up to 20 ng/mL. Values are shown as individual data points with mean ± SEM.

Analysis of individual subjects revealed the effects of tactile contact with an unfamiliar person on corticosterone levels. In most rabbits, this interaction was associated with an overall increase in hormone concentration compared to the control days (Figure 6). Only one rabbit showed no apparent change in corticosterone concentration (Figure 6D). The magnitude of the response varied among individual rabbits, with some exhibiting a more pronounced increase in corticosterone. Moreover, considerable variability was observed across the experimental measurements, and in some rabbits, notable fluctuations were also present during the control conditions, highlighting inter-individual differences.

**Figure 7:**
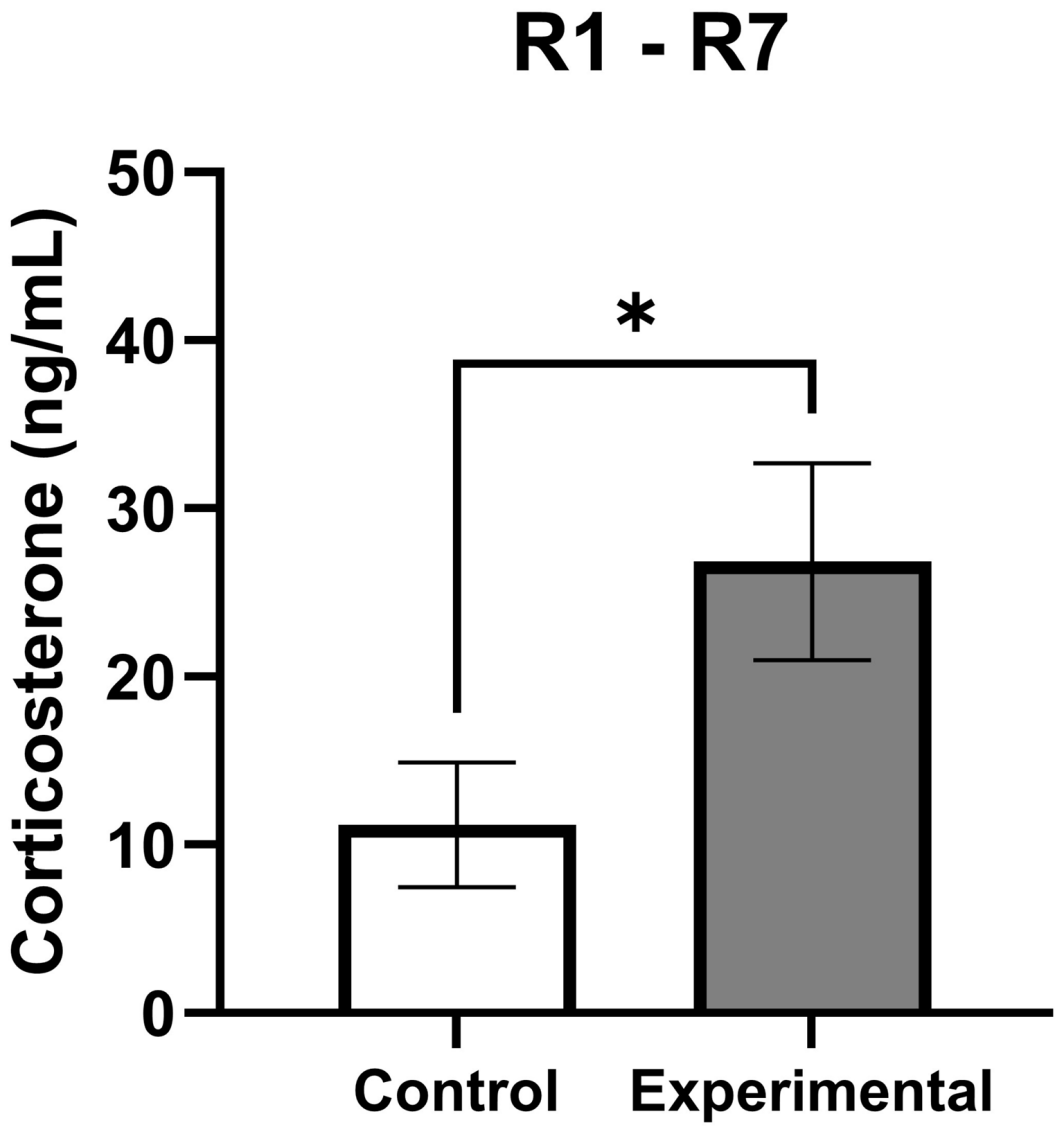
Mean corticosterone concentrations in rabbits. Comparison of mean corticosterone concentration (ng/mL) in rabbits (*n* = 7 R1–R7) during control (white bar) and experimental conditions (grey bar). For each rabbit, multiple measurements were performed (6 control and 5 experimental measurements). Data are presented as mean ± SEM. Statistical comparison between control and experimental conditions was performed using a paired Wilcoxon test; ^*^p < 0.05.

The group mean corticosterone concentration was significantly higher following contact with an unfamiliar person compared to control days without tactile contact with an unfamiliar person (*p* = 0.031) (Figure 7). The mean percentage increase in corticosterone concentration during the experimental stressor was +214.4 ± 74.1% relative to the control values.

### 3.2 Behavioral observation during tactile contact with an unfamiliar person

Behavioral tests assessed ear position, eye openness, and body configuration in rabbits that were exposed to physical contact with an unfamiliar person across five observation sessions.

**Figure 8:**
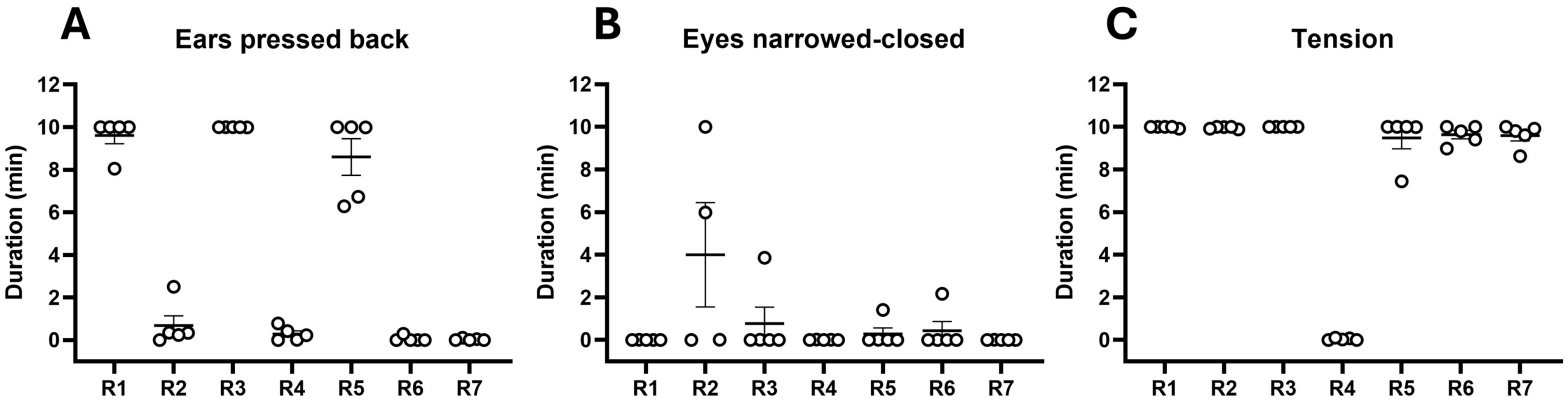
Behavioral observations. Individual behavior of rabbits (*n* = 7; R1–R7) during 10-min observations. Duration (min) of ears pressed back **(A)**, narrowed/closed eyes **(B)**, and body tension **(C)** is shown. Each dot represents a single measurement (min) from five independent 10-min observation session. Values are presented as individual points with mean ± SEM.

On average, rabbits held their ears pressed against their bodies for 4.18 min out of the 10-min observation period. Considerable inter-individual variability in the duration of ears pressed back was observed (Figure 8A). No significant differences were observed between observation sessions (*Q* = 7.09; *p* = 0.13).

Rabbits kept their eyes closed or partially closed for 0.69 min out of the 10-min observation period. Overall, inter-individual variability was generally low. Occasional deviations were observed in a small number of measurements, while the majority of rabbits showed consistent duration across observation sessions (Figure 8B). There were no significant differences between observation sessions (*F*_1.298, 7.465_ = 0.58; *p* = 0.51).

One rabbit clearly differed from the others in the time spent in tension (Figure 8C). When this individual was excluded, the duration of tension appeared comparable across all rabbits (see Supplementary Figure S1). Statistical comparison of the duration of tension across observation sessions revealed no significant differences (*Q* = 3.38; *p* = 0.50). For salivary corticosterone concentrations and behavioral parameters details in rabbits in control and experimental conditions (see Table 1).

**Table 1:** Salivary corticosterone concentrations and behavioral parameters in rabbits in control and experimental conditions.

Rabbit	Corticosterone (ng/mL)		Change relative to control (%)	Behavioral parameters (mean)		
	Control	Experimental		Ears pressed back	Eyes narrowed-closed	Body tension
R1	6.72 ± 1.8	37.39 ± 7.3	+456.4	9.61 ± 0.4	0.00 ± 0.0	9.98 ± 0.0
R2	17.59 ± 4.4	40.05 ± 7.2	+127.7	0.69 ± 0.5	4.00 ± 2.4	9.96 ± 0.0
R3	7.77 ± 3.0	47.39 ± 9.0	+509.9	10.00 ± 0.0	0.77 ± 0.8	10.00 ± 0.0
R4	30.19 ± 10.3	29.04 ± 8.5	−6	0.29 ± 0.1	0.01 ± 0.0	0.04 ± 0.0
R5	4.64 ± 0.7	12.50 ± 1.0	+169.4	8.60 ± 0.9	0.28 ± 0.3	9.49 ± 0.5
R6	3.81 ± 1.0	11.06 ± 0.7	+190.3	0.06 ± 0.1	0.43 ± 0.4	9.64 ± 0.2
R7	6.80 ± 1.0	10.42 ± 1.6	+53.2	0.03 ± 0.0	0.00 ± 0.0	9.59 ± 0.2

The table presents corticosterone levels (ng/mL) and behavioral parameters in individual rabbits (R1–R7). Corticosterone values are shown for control and experimental conditions (mean ± SEM) calculated from 6 measurements in control and 5 measurements in experimental conditions, together with the percentage change relative to the control. Behavioral parameters are expressed as mean scores across five 10-min measurements (mean ± SEM) and include ears pressed back, eyes narrowed or closed, and body tension.

### 3.3 Correlation between behavior observation and corticosterone levels

We found a positive correlation between body tension and corticosterone concentration (*r* = 0.82; *p* = 0.03). Moreover, there was an observed tendency toward a positive correlation between ears pressed back and corticosterone concentration (*r* = 0.71; *p* = 0.088). No correlation was found between eyes narrowed/closed and corticosterone concentration (*r* = 0.25; *p* = 0.58). For correlation details see Table 2.

**Table 2:** Correlation between percentage change in corticosterone level and behavioral parameters.

Behavioral parameters	*r*	*p*
Ears pressed back	0.71	0.088
Eyes narrowed-closed	0.25	0.58
Tension	0.82	**0.03** ^*^

Percentage difference in corticosterone concentration was calculated as the mean percent increase in corticosterone in response to tactile contact with an unfamiliar person. Behavioral parameters (ears pressed back, eyes narrowed/closed, and tension) represent the mean percentage of time spent in the given parameter calculated from five 10-min observations per animal; *r* represents Spearman’s correlation coefficient; ^*^p < 0.05.

## 4 Discussion

This study provides the first evidence that tactile interaction between a companion rabbit and an unfamiliar human—despite being gentle and non-invasive—clearly elicits acute stress responses, as demonstrated by both endocrine and behavioral indicators. Although an acute stress response is a natural adaptive mechanism, in pet-keeping conditions—and especially within animal-assisted interventions (AAI)—it may accumulate frequently due to repeated and potentially inappropriate handling. Such accumulation of acute stress responses can contribute to the development of chronic stress and allostatic load. The absence of an increase in corticosterone levels in response to handling suggests that this stimulus is unlikely to represent a significant risk for the development of allostatic load in rabbits (60, 61). It is important to emphasize that the rabbit remains a quintessential prey species and is also among the most recently domesticated companion animals (18, 62, 63). Consequently, its responses to challenging stimuli—and its subjective experience of such events—differ fundamentally from those of species with a long history of domestication, such as dogs (64, 65), horses (66, 67) or cats (68, 69).

Previous research illustrates clear species-specific differences in the endocrine markers typically used to assess stress response. In dogs and horses, stress-related studies most commonly measure cortisol, and these studies consistently report minimal physiological or behavioral responses during interactions with unfamiliar people. For example, Glenk et al. (70, 71), Kohoutková et al. (72), and McCullough et al. (73) found that dogs did not show increases in salivary cortisol in stress-related behaviors during such interactions or during animal-assisted interventions (AAI). Similarly, studies in horses demonstrate that contact with unfamiliar humans generally does not lead to notable changes in salivary cortisol or associated behavioral indicators (39, 74). In contrast, our study focused on rabbits, for which corticosterone—rather than cortisol—is the primary glucocorticoid and the hormone typically measured to assess stress. Here, salivary corticosterone concentrations increased significantly after the interaction, suggesting that, unlike dogs and horses, rabbits may exhibit a measurable endocrine stress response to tactile contact with unfamiliar humans. This pronounced hormonal response was accompanied by behavioral patterns that are commonly associated with discomfort or defensive arousal in rabbits: extended periods of body tension, ears pressed to the body. Importantly, corticosterone elevations positively correlated with key behavioral indicators, such as increased body tension and partially with ears pressed back, highlighting the concordance between physiological and behavioral stress markers.

This divergence from patterns observed in dogs and horses indicates that responses to unfamiliar humans cannot be generalized across domestic species and is likely to reflect species-specific vulnerabilities. Consequently, it is important to consider why rabbits, in particular, show a heightened physiological and behavioral response. One likely factor is smaller body size, which increases perceived predation risk, compared to horses, for example. Also, rabbits are highly sensitive to acute stress responses. Beside handling (75, 76), acute stress can also be readily triggered in companion rabbits by various factors, such as veterinary procedures (77), transport (78), or inappropriate housing (1, 14, 76, 79).

### 4.1 Corticosterone response of the rabbit to tactile contact with an unfamiliar person

The physiological responses observed in the present study support the hypothesis that salivary corticosterone concentrations increase following tactile interaction with an unfamiliar person. Most rabbits exhibited marked post-exposure elevations, with the group mean increasing by more than 200% relative to baseline values.

Although the establishment of true basal corticosterone levels in rabbits remains challenging, available evidence suggests that salivary corticosterone provides a reliable non-invasive indicator of acute HPA axis activation. Nevertheless, it is important to note that salivary glucocorticoids represent approximately 10% of circulating concentrations (80). However, this fraction corresponds to the biologically active, free hormone capable of diffusing from plasma into saliva (81) and bind to its receptors in target organs. Therefore, sampling saliva for corticosterone measurements seems the best noninvasive method to assess acute physical or psychological stressors, as proved by the measurable increase in salivary corticosterone observed in the present study.

The timing of saliva collection for assessing acute stress in rabbits remains uncertain, as the post-stress dynamics of salivary corticosterone have not yet been characterized in this species. Available evidence from related models is largely derived from invasive blood sampling in rats, where post-stress measurements have been obtained at 10, 25, 40, and 100 min (82) or within 15–55 min following stress exposure (83). Accordingly, in the present study, we collected saliva 20–25 min after the initial exposure to the stressor (an unfamiliar person). This sampling window was informed by canine studies of salivary cortisol, in which saliva is commonly collected approximately 20–25 min after an acute stressor (48, 49), including contexts such as separation from the caregiver (84) or hospitalization (48), enabling reliable detection of stress-related increases in glucocorticoids. Our results suggest that in rabbits, the saliva collection approximately 20–25 min post-stressor is an appropriate time window. Future studies should evaluate additional discrete time points (e.g., 15 and 30–40 min), but not repeated sampling after exposure to one stressor, as the sampling per se may induce stress.

While salivary corticosterone levels provide valuable insight into HPA axis activation, stress responses are inherently multifaceted. Future studies would benefit from the inclusion of additional physiological and neuroendocrine markers to better characterize the complexity of stress and emotional responses in rabbits. Potential candidates include catecholamines such as norepinephrine, reflecting sympathetic nervous system activation, as well as oxytocin, a neuropeptide associated with social bonding, affiliative behavior, and stress modulation, as used in other species (85, 86). As welfare is a broader concept than the mere absence of stress, it also includes positive experiences and the opportunity to meet species-specific needs. Therefore, assessing welfare is best done by combining behavioral and physiological indicators (87). The combined assessment of glucocorticoids and oxytocin may be particularly informative in studies of human–animal interactions, where tactile contact may simultaneously elicit stress-related and affiliative processes. In addition, incorporation of autonomic measures such as heart rate variability, respiratory rate, or peripheral temperature, alongside behavioral observations, could substantially enhance the interpretative value of future work.

### 4.2 Behavior observation of the rabbit during tactile contact with an unfamiliar person

In this study, three behavior indicators were observed: position of the ears, openness of the eyes, and body configuration. Ears pressed against body is a posture typically associated with emotional discomfort. During interaction with an unfamiliar person inter-individual variability was observed in the duration of this behavior, with some rabbits displaying it for nearly the entire observation, while others expressed it only briefly.

Although individuals differed in how strongly they expressed this behavior, its duration remained stable across sessions, indicating no signs of habituation. Eye closure or partial closure occurred only briefly and showed no meaningful variation between rabbits or across sessions, suggesting it was not a prominent component of their response. Rabbits also spent most of the interaction period in a tense body posture while positioned on the person’s lap; apart from one outlier, this behavior was comparable across individuals and likewise did not change over repeated sessions.

Based on this study’s results, the position of the ears appears to be a highly sensitive indicator of rabbits’ emotional state, which is in agreement with previously published studies on other species, for example, in dogs (88–90), pigs (91, 92), horses (93), and alpacas (94), where ears pressed back were associated with increased activation of the stress response. This indicator was also used in rabbits, especially as an indicator of discomfort and pain (95, 96), or in our previously published study, when rabbits interacted with children (5). It is also important to emphasize that ear retraction does not represent an intentional signaling behavior or a form of communication but rather constitutes a component of the emotional response coordinated by amygdala-driven neural circuits (88, 97, 98).

During the observation of tactile interaction with an unfamiliar person, the rabbits had their eyes normally open for most of the time. This indicator was monitored because stress and discomfort in animals are often manifested through changes in the eye area (5, 96) in connection with the activation of the autonomic nervous system. An indicator of the eye is also available for rabbits within so called “Facial grimace” scales; however, these were developed primarily for the detection of acute pain during veterinary procedures (10, 96), where discomfort is marked and specific facial action units, such as tightening around the eyes (eye narrowing), are clearly visible. These tools may therefore not be sufficiently sensitive for assessing mild stress during ordinary human–rabbit interaction. In some species, increasing stress intensity is also associated with increased visibility of the sclera (the “white of the eye”), as described in dogs, horses, cattle, pigs, and sheep (89, 99–104). In rabbits, a bulging appearance of the eyes has been described under extreme fear (105, 106), but this response was not observed in the individuals evaluated in our study. Indeed, in our previous study, Součková et al. (5) we assessed eye openness during animal-assisted intervention (AAI) and reported that when a rabbit was placed on a child’s lap, rabbits often closed/narrowed their eyes. In this study, this association was not confirmed, which may be related to differences in the course of the interaction: in the study by Součková et al. (5), the rabbits were in contact with school-aged children, in whom adherence to precise instructions and gentleness of handling may vary, and the children were also tasked with feeding the rabbits and touching them more intensively than in this study. This may have led to various results, underlying the fact that when rabbits are forced to interact with children on their lap, they are most probably under severe stress. To verify this hypothesis, further research is needed using stressors of varying intensity, for example, situations in which rabbits are not only gently stroked but are handled more firmly or manipulated in a more challenging manner. It is also important to consider that the type of stressor can influence rabbits’ behavioral responses. In line with Benato et al. (107), it is also necessary to interpret closed or partially closed eyes with caution: they may be associated with pain or discomfort, but can also occur during relaxation. Eye openness should therefore always be assessed in the context of overall behavior and together with other indicators, especially body posture and activity level, rather than as a single, isolated signal.

On average, rabbits remained in a tense body configuration for a substantial proportion of the human–animal interaction in our study. In our previously published study (5) freezing behavior of the rabbits was observed significantly more often in case when rabbits were on child’s lap, compared to when was alone, freezing behavior was defined as when the rabbit suddenly stiffened in a crouched position to the ground, had ears pressed back, and did not make any movement except for breathing. As in our understanding, freeze reaction is associated with a seriously threatening situation for the rabbit, in this study, we decided to adjust the observed behavior, and so we decided to observe rather body configuration in two possibilities: relaxed or tense. Tense body posture was associated with discomfort for the rabbit but not with a reaction when the rabbit was strongly scared. However, there is a discrepancy in the terminology used, mixing terms freezing and tension or even pressing self to the bottom. For example, Conway et al. (58) and Krall et al. (59) define freezing as “being immobile with a tense posture” and pressing down behavior as “pressing self into the bottom of the enclosure.” In practice, however, these behaviors may overlap. During pressing down, the rabbit is often also in a tense body posture, so “tense posture” alone is not a feature that reliably distinguishes between the two terms. Conway et al. (58) further notes that during pressing down, the muscles are tense and the rabbit is prepared to flee; similarly, freezing is described as a defensive state in which the animal is immobile but physiologically “prepared” for further action (108–110). The boundary between freezing, tense body posture, and pressing down is therefore subtle and depends on the context and the method of observation. For this reason, it is important to always specify precisely which specific signs are considered in a given study.

Taking all together to our understanding, tension represents a milder form of the freezing response, in which muscular rigidity is still present, but the overall stress load is lower. However, for full figure guidelines, regardless of whether we are talking about freeze or tension in prey species, a crouched posture might often be misinterpreted by lay observers as calmness or contentment (108). When people learn to observe this behavior, observing tension can be a good indicator of discomfort easily seen by humans, helping to strengthen the rabbit–human communication.

### 4.3 Correlation between behavior observation and corticosterone levels

To our knowledge, this study is the first to confirm a correlation between behavioral and physiological indicators, particularly corticosterone level in rabbits’ stress caused by tactile contact with unfamiliar humans. Increased body tension and ears pressed back against the body appear to be valuable indicators of acute stress. Increased body tension significantly correlated with elevated corticosterone levels and ears pressed back showed a tendency toward this correlation. Such a correlation was also observed in mice (111).

In behavior as well as in physiological measurements of corticosterone level, our results point to marked individual variability in stress response: whereas most individuals showed a pronounced increase in corticosterone, two rabbits exhibited a small or no change between control baseline and experimental level of corticosterone; moreover, they had less time ears pressed back to the body and spent less time in tension. This is an expected result since individual variability in animals’ responses to stress is reflected in differences in the magnitude and dynamics of the glucocorticoid response, with some animals responding only weakly to stressors, while others exhibit a pronounced hormonal reaction (51, 52). Both outline rabbits have been dominant leaders of the rabbits housed together. It is possible that animals in a dominant or leader position have permanently higher corticosterone levels and show smaller fluctuations between control and experimental measurements. According to Cavigelli et al. (82), individuals in a leading position remain constantly vigilant to defend their status. This finding confirms that stress reactivity in rabbits is not uniform and is likely associated with individual characteristics. Temperament and individual variability have become key biological concepts; current research demonstrates that understanding them requires examining the full spectrum of individual characteristics rather than relying solely on mean values (42, 51, 52). Studies on captive animals suggest that proactive or bold individuals generally exhibit a lower glucocorticoid stress response and that glucocorticoids may play an inhibitory role in proactive behavioral tendencies (112–114). Monitoring such individual differences in glucocorticoid levels and in behavioral responses to stressors is therefore crucial for understanding both animal welfare and adaptive potential (42, 115).

Taking everything together, most of the rabbits do not enjoy tactile interaction with an unfamiliar human while sitting on their lap. Further investigation of influencing factors such as personality, the exact manner of manipulation and handling of the rabbit during the interaction, and the age and sex of both the rabbits and the humans is needed.

Certain methodological and practical limitations must be acknowledged to contextualize our findings. The primary limitations of this study stem from the small number of animals included, which reduces the statistical power of the analyses. A further limitation is the use of exclusively intact female rabbits, which restricts the generalizability of the findings to the broader domestic rabbit population, as males and neutered individuals may exhibit different physiological and behavioral profiles of stress responsiveness. However, this was an intentional methodological choice to employ a highly homogeneous group of animals with comparable housing backgrounds, sex, and age. It remains unclear how rabbits perceive being lifted by a person, and whether their response differs depending on whether the handler is familiar or unfamiliar. In our study, rabbits were lifted by a familiar person, yet we cannot rule out the possibility that the lifting itself contributed to the acute stress reflected in elevated corticosterone levels. Singly housed rabbits were also less likely to struggle when lifted, a pattern previously reported in laboratory rabbits (75, 116) but not confirmed by Schepers et al. (117). Mullan and Main (118) suggest that rabbits handled less often may approach observers more readily because they have less reason to fear people. It is therefore surprising that the authors conclude that “confident handling” strengthens the human–rabbit bond and improves welfare.

## 5 Conclusion

This study is the first to systematically integrate behavioral and endocrine indicators of acute stress in domestic rabbits during interaction with an unfamiliar person, even though such situations commonly occur in household environments. Our findings clearly demonstrate that contact with an unfamiliar individual triggers a pronounced acute stress response with elevated corticosterone levels and behavioral indicators, such as ears pressed back and increased body tension.

This approach represents a significant methodological advancement, enabling a comprehensive assessment of rabbits’ acute emotional states in the context of human interaction and offering new insight into how domestic rabbits respond to situations involving direct physical contact with a human.

## Data Availability

The raw data supporting the conclusions of this article will be made available by the authors, without undue reservation.

## Supplementary material

SUPPLEMENTARY FIGURE 1 Behavioral observations (excluding outlier R4). Individual behavior of rabbits (*n* = 6; R1–R3, R5–R7) during 10-min observations. Duration (min) of ears pressed back **(A)**, narrowed/closed eyes **(B)**, and body tension **(C)**. Each dot represents a single measurement (min) from five independent 10-min observation sessions. Values are presented as individual points with mean ± SEM.
