# Supplementary figures and images for: When touch is stressful: acute endocrine and behavioral responses of domestic rabbits to unfamiliar human handling
Source: Front Vet Sci. 2026 Mar 6;13:1793812. doi: 10.3389/fvets.2026.1793812 (PMC13002407; doi:10.3389/fvets.2026.1793812)

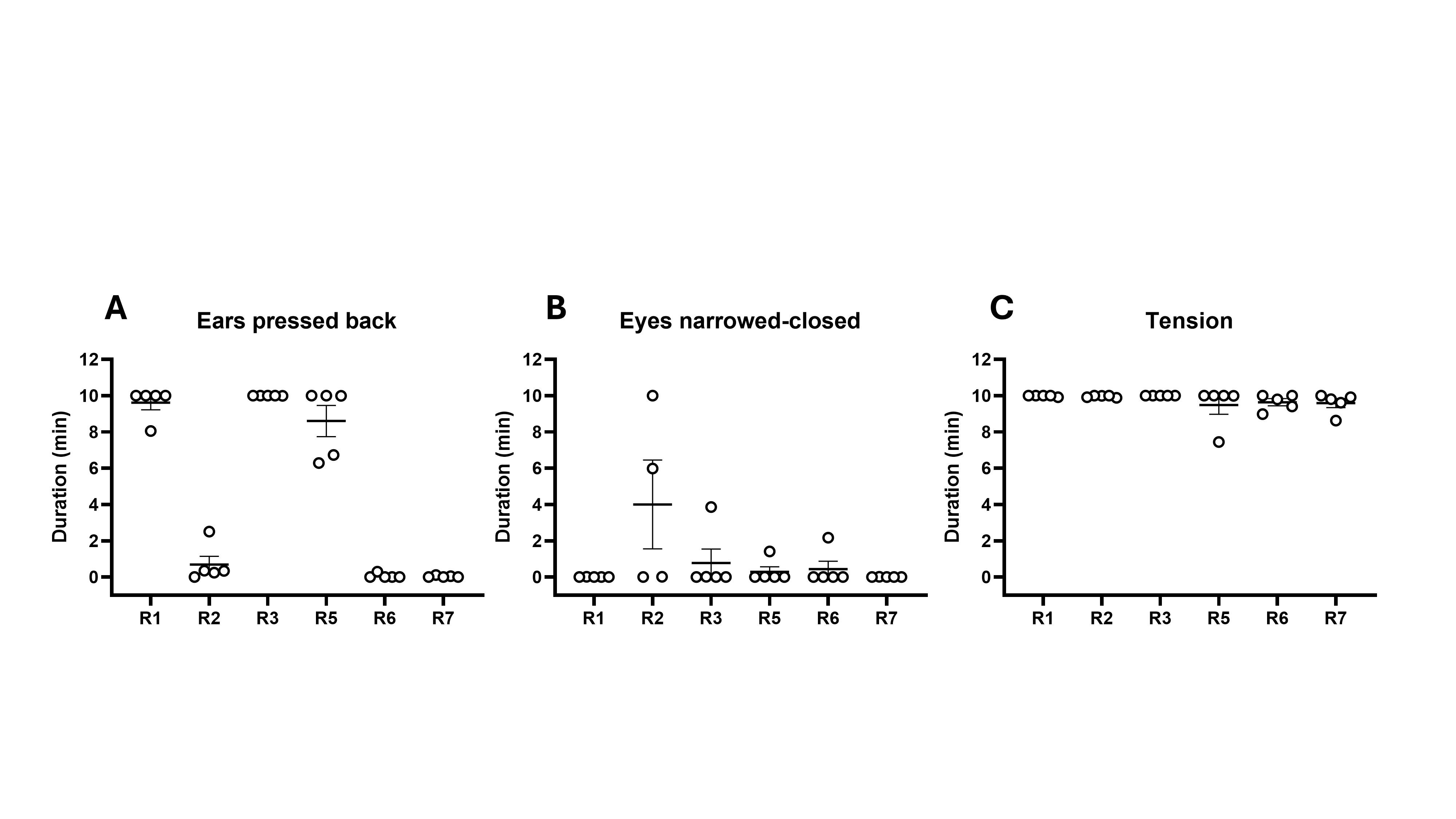

Supplement: SUPPLEMENTARY FIGURE 1 — Behavioral observations (excluding outlier R4). Individual behavior of rabbits (n = 6; R1–R3, R5–R7) during 10-min observations. Duration (min) of ears pressed back (A), narrowed/closed eyes (B), and body tension (C). Each dot represents a single measurement (min) from five independent 10-min observation sessions. Values are presented as individual points with mean ± SEM. [file Image_1.jpg]
