# Supplementary material for: When touch is stressful: acute endocrine and behavioral responses of domestic rabbits to unfamiliar human handling
Source: Front Vet Sci. 2026 Mar 6;13:1793812. doi: 10.3389/fvets.2026.1793812 (PMC13002407; doi:10.3389/fvets.2026.1793812)
Supplement: Supplementary file 2 [file Table_1.docx]

| Table S1. Dates of corticosterone collection | | | | | | |
| --- | --- | --- | --- | --- | --- | --- |
|  |  |  |  |  |  |  |
| **Control sampling dates** | | | | | | |
| Rabbit | Date1 | Date2 | Date3 | Date4 | Date5 | Date6 |
| 1 | 01.05.2025 | 07.05.2025 | 14.05.2025 | 19.05.2025 | 25.05.2025 | 01.06.2025 |
| 2 | 03.05.2025 | 10.05.2025 | 16.05.2025 | 23.05.2025 | 28.05.2025 | 03.06.2025 |
| 3 | 05.05.2025 | 11.05.2025 | 17.05.2025 | 21.05.2025 | 30.05.2025 | 04.06.2025 |
| 4 | 07.05.2025 | 13.05.2025 | 20.05.2025 | 25.05.2025 | 01.06.2025 | 07.06.2025 |
| 5 | 06.05.2025 | 12.05.2025 | 18.05.2025 | 24.05.2025 | 31.05.2025 | 06.06.2025 |
| 6 | 04.05.2025 | 09.05.2025 | 15.05.2025 | 22.05.2025 | 27.05.2025 | 05.06.2025 |
| 7 | 02.05.2025 | 08.05.2025 | 13.05.2025 | 20.05.2025 | 26.05.2025 | 02.06.2025 |
| **Experimental sampling dates** | | | | | |  |
| Rabbit | Date1 | Date2 | Date3 | Date4 | Date5 |  |
| 1 | 01.10.2025 | 08.10.2025 | 14.10.2025 | 19.10.2025 | 26.10.2025 |  |
| 2 | 14.10.2025 | 19.10.2025 | 26.10.2025 | 01.11.2025 | 06.11.2025 |  |
| 3 | 16.10.2025 | 22.10.2025 | 27.10.2025 | 02.11.2025 | 07.11.2025 |  |
| 4 | 09.10.2025 | 16.10.2025 | 21.10.2025 | 27.10.2025 | 03.11.2025 |  |
| 5 | 12.10.2025 | 18.10.2025 | 23.10.2025 | 30.10.2025 | 05.11.2025 |  |
| 6 | 04.10.2025 | 10.10.2025 | 17.10.2025 | 22.10.2025 | 28.10.2025 |  |
| 7 | 07.10.2025 | 12.10.2025 | 18.10.2025 | 25.10.2025 | 30.10.2025 |  |
